# Visceral fat score predicts diabetic kidney disease: analysis of 20 years of U.S. NHANES data

**DOI:** 10.1080/0886022X.2026.2650584

**Published:** 2026-04-07

**Authors:** Mengjiao Xu, Han Yan, Tian Gu, Qichao Yang, Yi Xue

**Affiliations:** Department of Endocrinology, Affiliated Wujin Hospital of Jiangsu University, Wujin Clinical College of Xuzhou Medical University, Changzhou, Jiangsu, China

**Keywords:** Precision medicine, diabetic kidney disease, noninvasive biomarkers, METS-VF, cardio-renal syndrome

## Abstract

Metabolic Score for Visceral Fat (METS-VF) serves as an innovative surrogate marker for evaluating visceral fat and associated cardiometabolic risks. This study aimed to explore the relationship between METS-VF and diabetic kidney disease (DKD). This cross-sectional study included diabetic patients aged 20 years or older who participated in the National Health and Nutrition Examination Surveys (NHANES). DKD was diagnosed in diabetic patients based on the presence of an impaired estimated glomerular filtration rate (eGFR <60 mL/min/1.73 m^2^) and/or albuminuria, defined as a urinary albumin-to-creatinine ratio (UACR) ≥30 mg/g. Logistic regression, subgroup analysis, restricted cubic spline (RCS), and mediation analysis were utilized. A total of 3871 diabetic patients were included in this study. The prevalence of DKD progressively increases with higher levels of METS-VF. Multivariate logistic regression analysis indicated that individuals in the highest METS-VF quartile exhibited adjusted odd ratios of 1.56 (95%CI: 1.28–1.91) for DKD, compared to those in the lowest quartile. METS-VF demonstrated superior discriminative performance with area under the curve (AUC) values of 58.0% for DKD, 56.1% for albuminuria, and 60.6% for low-eGFR compared to traditional obesity indices. RCS analysis reveal a distinct J-shaped relationship, with a turning point at 7.42. Subgroup analyses did not reveal specific populations, and hemoglobin A1c (HbA1c) was identified as a partial mediator in this association. METS-VF can serve as an epidemiological tool to quantify the effect of visceral fat on DKD risk. However, due to the cross-sectional design of this study, causality cannot be established, and further longitudinal research is necessary.

## Graphical Abstract

**Figure:**
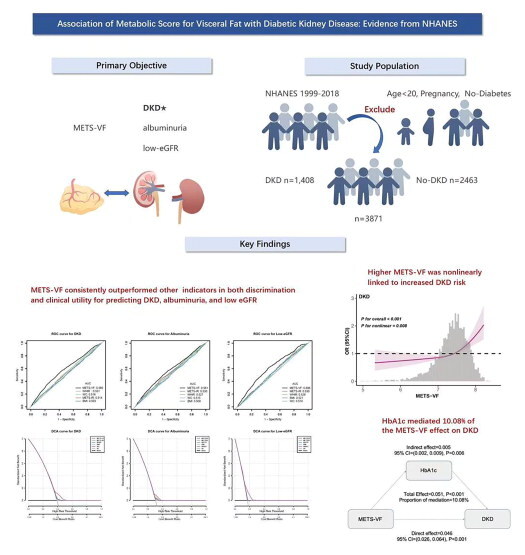

## 1. Introduction

Diabetic kidney disease (DKD), a severe complication of diabetes mellitus, is a leading cause of end-stage renal disease (ESRD) worldwide. In the United States alone, DKD affects approximately 40% of individuals diagnosed with diabetes [1]. DKD poses serious health risks to patients while also placing considerable economic and healthcare burdens on society [2].

Visceral fat has been increasingly recognized as a key modifiable factor among the diverse risk elements influencing the onset and progression of DKD [3–6]. Previous Mendelian randomization studies have also provided genetic evidence for a causal link between increased visceral fat and DKD risk [7]. This type of fat, deposited within the abdominal cavity around internal organs, contributes substantially to metabolic abnormalities such as insulin resistance, oxidative stress, and systemic inflammation [8–9]. Thus, the accurate measurement of visceral fat is essential for understanding its contribution to metabolic abnormalities [10]. While Magnetic Resonance Imaging (MRI) and Computed Tomography (CT) are considered the most reliable methods for measuring visceral fat distribution, their high expenses and technical requirements limit their use in wide-scale research [11]. To address these limitations, alternative methods for assessing visceral fat have been developed. Recently, the Metabolic Score for Visceral Fat (METS-VF) has gained attention as an innovative alternative [12]. By integrating metabolic score for insulin resistance (METS-IR), waist-to-height ratio (WHtR), age, and sex, this score outperforms other visceral fat proxies in terms of accuracy for assessing visceral fat accumulation and its related health risks [12–13]. Recent evidence also suggests that elevated METS-VF is strongly associated with heightened risks of diabetes, hypertension, cardiovascular diseases, and mortality [12,14–16]. Therefore, it becomes crucial to further explore the relationship between METS-VF and DKD risk.

While prior studies have explored BMI and waist circumference (WC) in DKD risk, none have systematically evaluated METS-VF in a nationally representative diabetic cohort. Using the National Health and Nutrition Examination Surveys (NHANES) database, this study aimed to examine the link between METS-VF and the prevalence of DKD in a representative sample of the national population.

## 2. Materials and methods

### 2.1. Study population

NHANES (https://wwwn.cdc.gov/nchs/nhanes/), conducted by the National Center for Health Statistics (NCHS) at the Centers for Disease Control and Prevention, utilized a randomized, stratified, and multi-stage sampling design to guarantee national representation. Participants in NHANES took part in physical evaluations, responded to questionnaires, and underwent laboratory testing. Ethical approval for NHANES protocols was granted by the NCHS Ethics Review Board, with participants providing written informed consent (https://www.cdc.gov/nchs/nhanes/about/erb.html). The analysis incorporated data collected from 10 NHANES cycles between 1999 and 2000 and 2017–2018. Participants excluded were those under 20 years old, pregnant, non-diabetic, or lacking information on METS-VF, urinary albumin-to-creatinine ratio (UACR), or estimated glomerular filtration rate (eGFR). Following these exclusions, 3871 eligible participants with diabetes were included in the study.

### 2.2. Calculation of METS-VF

METS-VF represents an index developed to evaluate visceral fat accumulation and its association with cardiometabolic health [12]. The calculation of METS-VF is based on the formula: METS-VF = 4.466 + 0.011 × [Ln (METS-IR)]³ + 3.239 × [Ln (WHtR)]³ + 0.319 × (sex) + 0.594 × [Ln (age, years)] (“male” = 1, “female” = 0) [12]. METS-IR is determined using the equation: METS-IR = Ln [(2 × fasting plasma glucose, mg/dL) + triglyceride, mg/dL] × body mass index, kg/m^2^/[Ln high-density lipoprotein cholesterol, mg/dL] [17]. WHtR is calculated as WC (cm) divided by height (cm).

### 2.3. Definition of DKD

Diabetes was identified based on self-reported medical history, fasting plasma glucose (FPG) levels of ≥126 mg/dL, hemoglobin A1c (HbA1c) values of ≥6.5%, or the administration of antidiabetic drugs. The UACR was utilized to evaluate the presence of albuminuria (UACR ≥30 mg/g), while the eGFR was calculated using the Chronic Kidney Disease Epidemiology Collaboration Equation (CKD-EPI), which includes parameters such as age, sex, ethnicity, and serum creatinine (SCr) [18]. DKD was diagnosed in individuals with diabetes if the UACR was ≥30 mg/g and/or the eGFR was <60 mL/min/1.73 m^2^.

### 2.4. Covariates

Demographic and other covariate data, including age, sex, ethnicity, family income to poverty ratio (PIR), education level, smoking status, hypertension, congestive heart failure (CHF), coronary artery disease (CHD), body mass index (BMI), WC, height, FPG, HbA1c, triglycerides (TG), total cholesterol (TC), high-density lipoprotein cholesterol (HDL-c), low-density lipoprotein cholesterol (LDL-c), and serum uric acid (SUA), were collected. Ethnicity classifications comprised Mexican American, Other Hispanic, Non-Hispanic White, Non-Hispanic Black, and Other Race. Education was divided into three categories: less than high school, high school, and college or more. Smokers were defined as both current and former smokers. BMI was classified as <25, 25–29.9, and ≥30 kg/m^2^. WHtR was derived as WC divided by height. Hypertension was defined using systolic blood pressure ≥140 mmHg, diastolic blood pressure ≥90 mmHg, a self-reported medical history, or antihypertensive medication use. CHF and CHD classifications were based on self-reported histories. Detailed measurement guidance is available through NHANES at https://wwwn.cdc.gov/nchs/nhanes/.

### 2.5. Statistical analysis

Population-weighted descriptive statistics were calculated in this study (https://wwwn.cdc.gov/nchs/nhanes/analyticguidelines.aspx). Continuous variables were reported as medians with interquartile ranges, and categorical variables as counts with weighted percentages. Missing data was assumed to follow a Missing at Random (MAR) distribution and handled using random forest-based imputation through the missForest R package. The algorithm utilized 10 maximum iterations with 100 trees per forest. Out-of-bag error assessment demonstrated adequate imputation quality (NRMSE = 0.0004, PFC = 0.092). The Kruskal-Wallis test and the Rao-Scott chi-square test were used to compare variables among groups. Logistic regression models were used to investigate associations between METS-VF and DKD, albuminuria (UACR ≥30 mg/g), and low-eGFR (eGFR < 60 mL/min/1.73 m^2^) risk, with three models used for different levels of covariate adjustment (Model 1, without covariates adjusted; Model 2, with adjustments for ethnicity, PIR, education level, marital status, and smoking status; Model 3, with further adjustments for hypertension, CHD, CHF, TC, LDL, and SUA based on Model 2). Restricted cubic spline (RCS) models were constructed using the rms package to evaluate the non-linear association between METS-VF and DKD risk. Knots were placed at the 5th, 35th, 65th, and 95th percentiles, with the 50th percentile serving as the reference value. Non-linearity was assessed using Wald tests *via* the anova() function. The threshold was identified by testing all values and selecting the one maximizing likelihood. A two-piecewise regression model was used to examine DKD risk associated with METS-VF on either side of the threshold. Receiver Operating Characteristic (ROC) and Decision Curve Analysis (DCA) were used to evaluate the discriminative performance of METS-VF for DKD risk. Subgroup analyses were performed based on covariates. Additionally, mediating effect analysis was also performed. The statistical analyses in this research were performed by the Empower software (http://www.empowerstats.com) and R 4.2.1 (http://www.R-project.org). A two-side *p* value <0.05 was considered statistically significant.

## 3. Results

### 3.1. Basic characteristics of diabetic population

The study analyzed 3871 individuals with diabetes (median age: 63 years; interquartile range: 52–71 years), comprising 773 Mexican Americans (weighted 9.45%), 924 non-Hispanic Black individuals (weighted 13.75%), 1429 non-Hispanic White individuals (weighted 62.44%), 387 other Hispanics (weighted 6.08%), and 358 individuals of other races (weighted 8.29%) (Table 1). DKD participants were typically older, unmarried, less educated, and had lower PIR, height, LDL-c, and eGFR levels (*p* < 0.01). Conversely, DKD individuals demonstrated elevated levels of FPG, HbA1c, TG, SUA, and UACR and had a greater prevalence of hypertension, CHD, and CHF (*p* < 0.01). METS-VF levels were substantially higher in DKD individuals compared to the non-DKD group (*p* < 0.001).

**Table 1.** Baseline characteristics stratified by the presence or absence of DKD.

Characteristic	Overall (3871)	Non-DKD (*n* = 2356)	DKD (*n* = 1515)	*p* value
Age (years)	63.00 (52.00–71.00)	60.00 (50.00–68.00)	67.00 (58.00–76.00)	<0.001
Sex, *n*%				0.425
Female	1822 (47.75%)	1121 (48.07%)	701 (47.16%)	
Male	2049 (52.25%)	1235 (51.93%)	814 (52.84%)	
Ethnicity, *n*%				0.010
Mexican American	773 (9.45%)	466 (9.29%)	307 (9.75%)	
Other hispanic	387 (6.08%)	258 (6.47%)	129 (5.33%)	
Non-hispanic white	1429 (62.44%)	832 (62.60%)	597 (62.13%)	
Non-hispanic black	924 (13.75%)	565 (13.54%)	359 (14.14%)	
Other race	358 (8.29%)	235 (8.09%)	123 (8.65%)	
PIR	1.92 (1.13–3.30)	2.03 (1.16–3.51)	1.78 (1.10–2.98)	<0.001
Education level, *n*%				<0.001
Less than High school	1405 (23.77%)	788 (21.60%)	617 (27.87%)	
High school	894 (25.99%)	555 (25.40%)	339 (27.11%)	
Some college or above	1572 (50.24%)	1013 (53.01%)	559 (45.02%)	
Married, *n*%				0.002
No	1671 (40.36%)	970 (38.39%)	701 (44.08%)	
Yes	2200 (59.64%)	1386 (61.61%)	814 (55.92%)	
Smoking status, *n*%				0.075
No	1873 (48.68%)	1167 (49.68%)	706 (46.81%)	
Yes	1998 (51.32%)	1189 (50.32%)	809 (53.19%)	
BMI (kg/m^2^)	30.75 (26.94–35.64)	30.79 (27.08–35.60)	30.74 (26.80–35.70)	0.737
WC (cm)	106.70 (97.30–117.70)	106.40 (97.20–117.20)	107.20 (97.50–118.50)	0.058
Height (cm)	166.30 (158.95–173.70)	166.60 (159.50–174.30)	165.50 (158.20–172.90)	<0.001
FPG (mg/dL)	138.00 (121.00–175.00)	135.00 (120.00–167.00)	144.00 (122.00–189.95)	<0.001
HbA1c (%)	6.70 (6.10–7.90)	6.60 (6.00–7.60)	6.90 (6.20–8.30)	<0.001
TG (mg/dL)	136.00 (95.00–199.00)	134.00 (92.00–189.00)	141.00 (99.00–212.50)	0.002
TC (mg/dL)	184.00 (158.00–215.00)	186.00 (160.00–215.00)	182.00 (154.00–216.00)	0.405
HDL (mg/dL)	46.00 (39.00–56.00)	46.00 (40.00–56.00)	46.00 (39.00–56.00)	0.434
LDL (mg/dL)	105.62 (82.00–133.00)	108.00 (84.00–134.00)	103.00 (79.00–131.00)	0.004
SUA (mg/dL)	5.70 (4.70–6.80)	5.50 (4.60–6.50)	6.00 (5.00–7.30)	<0.001
UACR (mg/g)	12.95 (6.75–43.42)	8.54 (5.57–13.74)	64.17 (32.12–193.15)	<0.001
eGFR (mL/min/1.73m^2^)	88.03 (68.75–102.75)	93.50 (80.44–105.46)	68.39 (50.01–95.33)	<0.001
METS-VF	7.43 (7.15–7.66)	7.38 (7.12–7.61)	7.49 (7.21–7.72)	<0.001
Hypertension, *n*%				<0.001
No	1172 (31.52%)	876 (37.37%)	296 (20.46%)	
Yes	2699 (68.48%)	1480 (62.63%)	1219 (79.54%)	
CHD, *n*%				<0.001
No	3511 (90.38%)	2201 (92.91%)	1310 (85.60%)	
Yes	360 (9.62%)	155 (7.09%)	205 (14.40%)	
CHF, *n*%				<0.001
No	3550 (92.59%)	2250 (95.84%)	1300 (86.45%)	
Yes	321 (7.41%)	106 (4.16%)	215 (13.55%)	

Abbreviations: PIR: family income-to-poverty ratio, BMI: body mass index, WC: waist circumference, FPG: fasting plasma glucose, HbA1c: hemoglobin A1c, TG: triglyceride, TC: total cholesterol, HDL-c: high-density lipoprotein cholesterol, LDL-c: low-density lipoprotein cholesterol, SUA: serum uric acid, UACR: urinary albumin-to-creatinine ratio, eGFR: estimated glomerular filtration rate, METS-VF: Metabolic Score for Visceral Fat, CHD: coronary heart disease, CHF: congestive heart failure.

Based on METS-VF levels, participants were stratified into four quartiles (Q1, Q2, Q3, and Q4) (Table 2). Compared to Q1, participants in higher quartiles were significantly older, more likely to be male, had higher PIR, were more frequent smokers, and exhibited higher levels of BMI and WC but lower height (*p* < 0.001). Biochemical indicators like FPG, HbA1c, TG, TC, HDL-c, LDL-c, SUA, UACR, and eGFR varied significantly among different quartiles (*p* < 0.001). Moreover, the prevalence of DKD progressively increased with higher METS-VF levels (*p* < 0.001).

**Table 2.** Baseline data categorized by quartile levels of METS-VF.

Characteristic	Q1	Q2	Q3	Q4	*p* value
Age (years)	58.00 (46.00–67.00)	61.00 (51.00–70.00)	64.00 (55.00–72.00)	66.00 (58.00–74.00)	<0.001
Sex, *n*%					<0.001
Female	543 (58.02%)	560 (57.86%)	514 (56.14%)	205 (20.81%)	
Male	425 (41.98%)	407 (42.14%)	454 (43.86%)	763 (79.19%)	
Ethnicity, *n*%					<0.001
Mexican American	179 (10.02%)	216 (10.76%)	206 (9.71%)	172 (7.47%)	
Other hispanic	98 (7.59%)	95 (6.45%)	110 (6.04%)	84 (4.39%)	
Non-hispanic white	277 (50.49%)	320 (60.57%)	355 (64.50%)	477 (73.20%)	
Non-hispanic black	247 (16.59%)	240 (14.83%)	241 (13.67%)	196 (10.18%)	
Other race	167 (15.32%)	96 (7.39%)	56 (6.08%)	39 (4.76%)	
PIR	1.99 (1.19–3.49)	1.79 (1.04–3.14)	1.79 (1.11–3.06)	2.07 (1.23–3.44)	<0.001
Education level, *n*%					0.086
Less than high school	314 (24.56%)	365 (23.88%)	363 (22.69%)	363 (23.98%)	
High school	225 (25.43%)	229 (25.24%)	228 (27.30%)	212 (25.93%)	
Some college or above	429 (50.01%)	373 (50.88%)	377 (50.00%)	393 (50.10%)	
Married, *n*%					0.066
No	442 (44.86%)	420 (42.06%)	424 (37.42%)	385 (37.47%)	
Yes	526 (55.14%)	547 (57.94%)	544 (62.58%)	583 (62.53%)	
Smoking status, *n*%					<0.001
No	540 (55.80%)	502 (50.61%)	455 (45.95%)	376 (42.97%)	
Yes	428 (44.20%)	465 (49.39%)	513 (54.05%)	592 (57.03%)	
BMI (kg/m^2^)	25.13 (23.02–27.72)	29.56 (27.15–32.91)	32.21 (29.45–36.50)	36.60 (32.72–42.07)	<0.001
WC (cm)	91.80 (86.57–96.80)	102.80 (98.10–108.75)	110.70 (105.57–117.50)	122.70 (115.50–132.00)	<0.001
Height (cm)	165.30 (158.28–172.83)	164.20 (157.75–172.15)	164.90 (157.70–171.95)	170.30 (163.78–176.20)	<0.001
FPG (mg/dL)	134.00 (116.00–171.00)	134.00 (119.00–168.50)	138.00 (121.00–177.00)	144.00 (125.00–182.30)	<0.001
HbA1c (%)	6.60 (5.90–7.80)	6.70 (6.00–7.90)	6.80 (6.20–7.90)	6.90 (6.30–7.80)	<0.001
TG (mg/dL)	113.00 (80.00–173.00)	133.00 (93.00–194.50)	143.00 (101.00–205.00)	151.00 (109.00–222.00)	<0.001
TC (mg/dL)	192.00 (164.00–222.25)	190.00 (162.00–219.50)	182.00 (158.00–213.00)	173.00 (149.00–204.00)	<0.001
HDL (mg/dL)	52.00 (43.00–65.00)	48.00 (41.00–58.00)	45.00 (39.00–55.00)	42.00 (35.00–48.00)	<0.001
LDL (mg/dL)	110.00 (86.00–138.03)	111.00 (85.00–138.00)	104.00 (81.00–130.00)	98.00 (76.00–124.00)	<0.001
SUA (mg/dL)	5.15 (4.30–6.10)	5.50 (4.60–6.50)	5.85 (4.90–6.90)	6.30 (5.30–7.40)	<0.001
UACR (mg/g)	11.90 (6.34–34.43)	11.64 (6.34–30.94)	12.95 (7.08–45.25)	15.84 (7.78–65.21)	<0.001
eGFR (mL/min/1.73m^2^)	95.33 (76.53–108.84)	91.36 (74.55–104.96)	87.04 (65.86–100.26)	79.09 (60.70–94.69)	<0.001
METS-VF	6.91 (6.65–7.05)	7.30 (7.23–7.36)	7.54 (7.48–7.59)	7.81 (7.73–7.92)	<0.001
Hypertension, *n*%					<0.001
No	440 (48.61%)	312 (34.39%)	215 (23.58%)	205 (20.76%)	
Yes	528 (51.39%)	655 (65.61%)	753 (76.42%)	763 (79.24%)	
CHD, *n*%					<0.001
No	920 (96.04%)	895 (93.21%)	875 (89.03%)	821 (83.57%)	
Yes	48 (3.96%)	72 (6.79%)	93 (10.70%)	147 (16.43%)	
CHF, *n*%					<0.001
No	928 (96.88%)	915 (95.95%)	871 (90.18%)	836 (87.86%)	
Yes	40 (3.12%)	52 (4.05%)	97 (9.82%)	132 (12.14%)	
DKD, *n*%					<0.001
No	646 (72.23%)	648 (71.52%)	573 (62.73%)	489 (55.92%)	
Yes	322 (27.77%)	319 (28.48%)	395 (37.27%)	479 (44.08%)	

Abbreviations: PIR: family income-to-poverty ratio; BMI: body mass index; WC: waist circumference; FPG: fasting plasma glucose; HbA1c: hemoglobin A1c; TG: triglyceride; TC: total cholesterol; HDL-c: high-density lipoprotein cholesterol; LDL-c: low-density lipoprotein cholesterol; SUA: serum uric acid; UACR: urinary albumin-to-creatinine ratio; eGFR: estimated glomerular filtration rate; METS-VF: Metabolic Score for Visceral Fat; CHD: coronary heart disease; CHF: congestive heart failure.

### 3.2. METS-VF and DKD, low-eGFR, and albuminuria risk

Table 3 presents the association between METS-VF levels and the risks of DKD, albuminuria, and low eGFR risk. Individuals were divided into quartiles (Q1–Q4) based on METS-VF levels, with Q1 serving as the reference category. Unadjusted odd ratios (ORs) and 95% confidence intervals (CIs) for DKD risk were observed as 1.00 (reference), 0.99 (0.82–1.19), 1.38 (1.15–1.66), and 1.97 (1.64–2.36). For albuminuria, the ORs were 1.00 (reference), 0.91 (0.75–1.12), 1.24 (1.02–1.51), and 1.64 (1.36–1.99), while for low-eGFR, the ORs across quartiles were 1.00 (reference), 1.10 (0.84–1.45), 1.77 (1.37–2.28), and 2.37 (1.86–3.03). Model 2 also yielded a significant positive trend in the risks of DKD, low-eGFR, and albuminuria as METS-VF increased (*p* for trend < 0.001). Fully adjusted in Model 3, the ORs for DKD across Q1–Q4 were 1.00 (reference), 0.88 (0.72–1.08), 1.10 (0.91–1.34), and 1.56 (1.28–1.91). The ORs for albuminuria risk were 1.00 (reference), 0.83 (0.67–1.02), 1.06 (0.86–1.30), and 1.48 (1.20–1.82), while low-eGFR risk had ORs of 1.00 (reference), 1.01 (0.76–1.34), 1.37 (1.04–1.80), and 1.70 (1.29–2.23). Furthermore, a positive association was observed between METS-VF, analyzed as a continuous variable, and the risks of DKD and albuminuria, as well as low eGFR (*p* < 0.001).

**Table 3.** Logistic regression model of METS-VF and DKD, albuminuria, and low-eGFR.

	Model 1	Model 2	Model 3
	OR (95%CI), p value		
DKD			
METS-VF	1.94 (1.65, 2.28) <0.001	1.92 (1.63, 2.26) <0.001	1.57 (1.32, 1.87) <0.001
Quantiles			
Q1	Ref.	Ref.	Ref.
Q2	0.99 (0.82, 1.19) 0.897	0.96 (0.79, 1.16) 0.685	0.88 (0.72, 1.08) 0.224
Q3	1.38 (1.15, 1.66) <0.001	1.35 (1.12, 1.63) 0.002	1.10 (0.91, 1.34) 0.331
Q4	1.97 (1.64, 2.36) <0.001	1.93 (1.59, 2.33) <0.001	1.56 (1.28, 1.91) <0.001
*p* for trend	<0.001	<0.001	<0.001
Albuminuria			
METS-VF	1.66 (1.40, 1.96) <0.001	1.72 (1.44, 2.05) <0.001	1.49 (1.24, 1.79) <0.001
Quantiles			
Q1	Ref.	Ref.	Ref.
Q2	0.91 (0.75, 1.12) 0.388	0.90 (0.73, 1.10) 0.292	0.83 (0.67, 1.02) 0.083
Q3	1.24 (1.02, 1.51) 0.032	1.23 (1.01, 1.50) 0.044	1.06 (0.86, 1.30) 0.577
Q4	1.64 (1.36, 1.99) <0.001	1.72 (1.41, 2.10) <0.001	1.48 (1.20, 1.82) <0.001
*p* for trend	<0.001	<0.001	<0.001
Low-eGFR			
METS-VF	2.65 (2.11, 3.34) <0.001	2.46 (1.95, 3.12) <0.001	1.90 (1.49, 2.43) <0.001
Quantiles			
Q1	Ref.	Ref.	Ref.
Q2	1.10 (0.84, 1.45) 0.484	1.08 (0.82, 1.42) 0.583	1.01 (0.76, 1.34) 0.963
Q3	1.77 (1.37, 2.28) <0.001	1.73 (1.34, 2.24) <0.001	1.37 (1.04, 1.80) 0.023
Q4	2.37 (1.86, 3.03) <0.001	2.19 (1.70, 2.83) <0.001	1.70 (1.29, 2.23) <0.001
*p* for trend	<0.001	<0.001	<0.001

Abbreviations: METS-VF: Metabolic Score for Visceral Fat; DKD: diabetic kidney disease; Low-eGFR: low-estimated glomerular filtration rate.

OR: odds ratio. 95% CI: 95% confidence interval.

Model1: Non-adjusted.

Model 2: adjusted for ethnicity, PIR, education level, marital status, and smoking status.

Model 3: adjusted for ethnicity, PIR, education level, marital status, smoking status, hypertension, CHD, CHF, TC, LDL, and SUA.

### 3.3. ROC and DCA analysis

**Figure 1.**
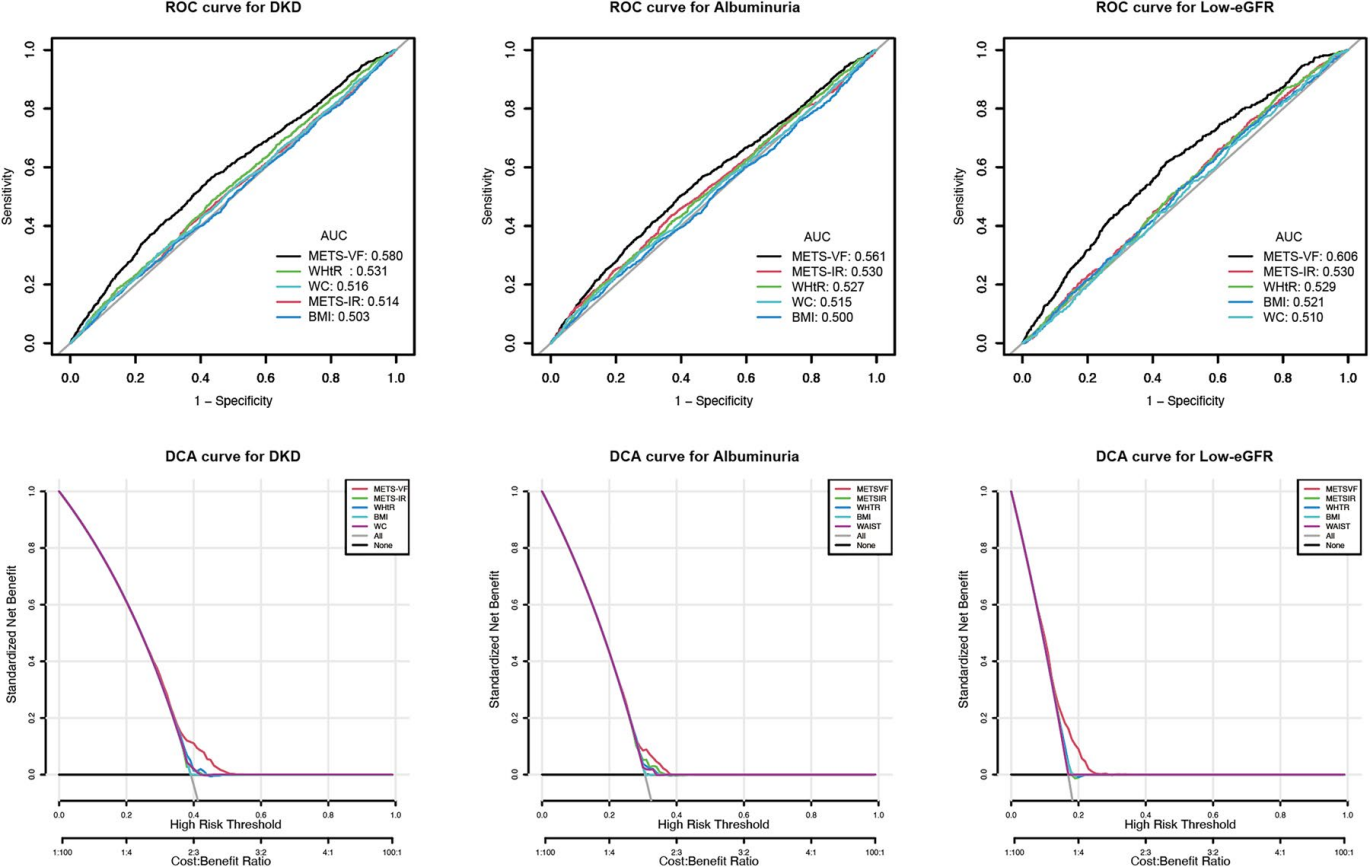
The results of ROC and DCA analysis. Abbreviations: ROC: receiver operating characteristic; DCA: decision curve analysis; AUC: area under the curve; METS-VF: Metabolic Score for Visceral Fat; METS-IR: Metabolic Score for Insulin Resistance; WHtR: waist-to-height ratio; BMI: body mass index; WC: waist circumference; DKD: diabetic kidney disease; eGFR: estimated glomerular filtration rate.

Figure 1 illustrates the results of ROC and DCA analyses comparing the discriminative capacities of the METS-VF, WHtR, METS-IR, BMI, and WC for DKD and albuminuria and low-eGFR risk. According to the area under the curve (AUC) values for DKD risk, METS-VF achieved 58.0%, WHtR 53.1%, METS-IR 51.4%, BMI 50.3%, and WC 51.6%. For albuminuria, the AUC values were METS-VF (56.1%), WHtR (52.7%), METS-IR (53.0%), BMI (50.0%), and WC (51.5%), while for low-eGFR, they were METS-VF (60.6%), WHtR (52.9%), METS-IR (53.0%), BMI (52.1%), and WC (51.0%) (Table 4). Moreover, DCA analysis revealed that the METS-VF showed higher discriminative ability than WHtR, METS-IR, BMI, and WC.

**Table 4.** Specific ROC analysis results for DKD, albuminuria, and low-eGFR.

ROC	AUC	95%CI lower	95%CI upper	Specificity	Sensitivity
DKD					
METS-VF	0.5805	0.5620	0.5989	0.5772	0.5551
METS-IR	0.5142	0.4954	0.5329	0.6367	0.4007
WHtR	0.5306	0.5120	0.5492	0.5671	0.4845
BMI	0.5028	0.4841	0.5216	0.8115	0.2132
WC	0.5156	0.4969	0.5343	0.7322	0.3050
Albuminuria					
METS-VF	0.5614	0.5416	0.5812	0.6086	0.4970
METS-IR	0.5300	0.5099	0.5500	0.6291	0.4370
WHtR	0.5273	0.5075	0.5470	0.7440	0.3018
BMI	0.4997	0.4797	0.5197	0.8032	0.2223
WC	0.5150	0.4951	0.5349	0.7292	0.3085
Low-eGFR					
METS-VF	0.6064	0.583	0.6298	0.5571	0.6189
METS-IR	0.5299	0.5063	0.5536	0.3988	0.6616
WHtR	0.5286	0.5051	0.5521	0.1975	0.8659
BMI	0.5205	0.4966	0.5444	0.5204	0.5274
WC	0.5096	0.4858	0.5333	0.3621	0.6707

Abbreviations: ROC: receiver operating characteristic; AUC: area under the curve; 95% CI: 95% confidence interval; DKD: diabetic kidney disease; METS-VF: Metabolic Score for Visceral Fat; METS-IR: Metabolic Score for Insulin Resistance; WHtR: waist-to-height ratio; BMI: body mass index; WC: waist circumference; eGFR: estimated glomerular filtration rate.

### 3.4. Nonlinear trends between METS-VF and DKD

**Figure 2.**
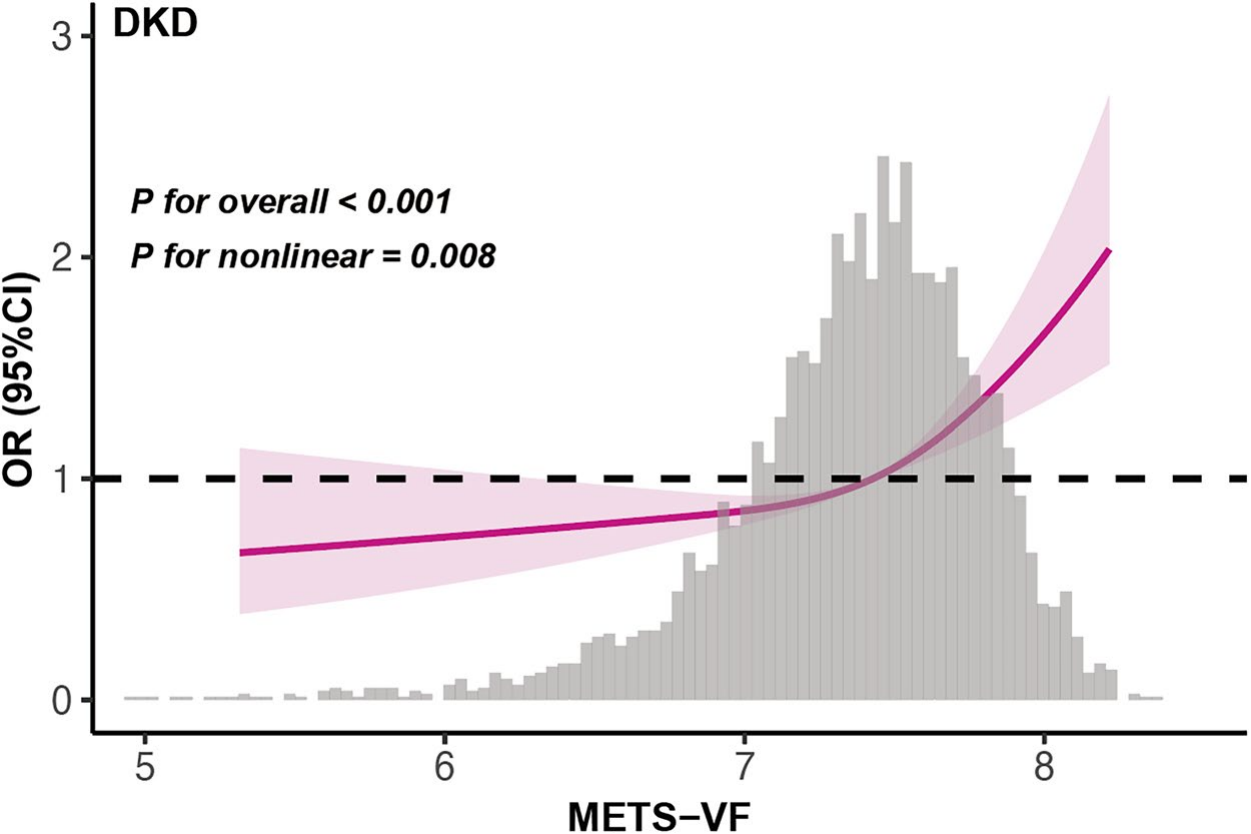
The results of RCS analysis.

The RCS analysis revealed a nonlinear relationship between METS-VF and the risk of DKD, highlighting a J-shaped association after adjusting for confounding factors (*p* for nonlinearity = 0.008) (Figure 2). A two-segment regression analysis identified a breakpoint at 7.42 for METS-VF levels in the total population (Table 5). Below this threshold, the risk of DKD gradually increased, with an OR of 1.18 (95%CI: 0.92–1.51). Above the threshold, the risk of DKD heightened more sharply, with an OR of 2.77 (95% CI: 1.85–4.16).

**Table 5.** Threshold effect analysis of METS-VF on DKD.

DKD	OR (95% CI) *p* value
Fitting by standard regression model	1.57 (1.32, 1.87) <0.001
Fitting by two-piecewise regression model	
Inflection point	7.42
OR1(METS-VF <7.42)	1.18 (0.92, 1.51) 0.183
OR2 (METS-VF ≥7.42)	2.77 (1.85, 4.16) <0.001
OR2/OR1	2.35 (1.36, 4.06) 0.002
*p* for Log-likelihood ratio	0.002

OR: odds ratio. 95% CI: 95% confidence interval.

adjusted for ethnicity, PIR, education level, marital status, smoking status, hypertension, CHD, CHF, TC, LDL, and SUA.

### 3.5. Subgroup analyses

**Figure 3.**
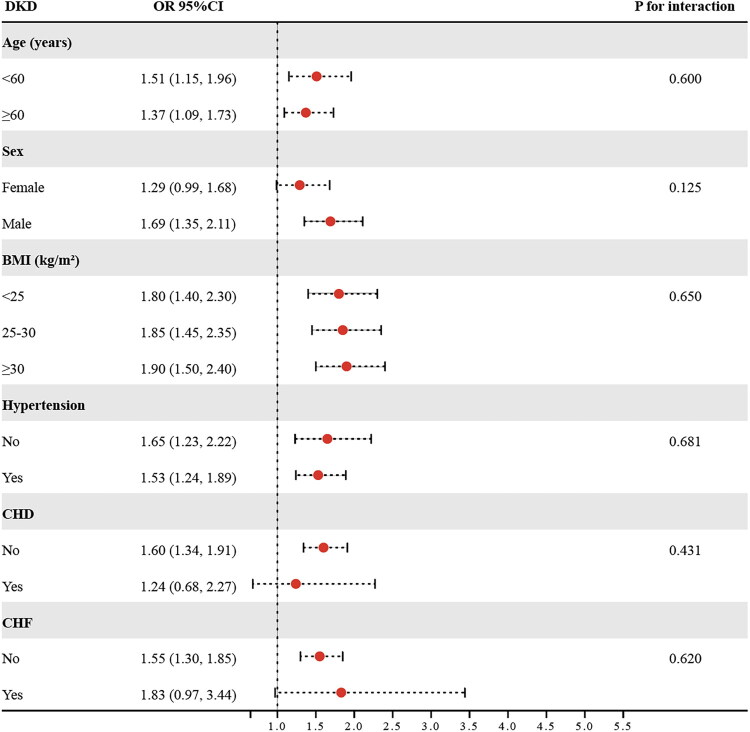
The results of subgroup analyses. Abbreviations: DKD: diabetic kidney disease; BMI: body mass index; CHD: coronary heart disease; CHF: congestive heart failure; OR: odds ratio; 95%CI: 95% confidence interval.

Subgroup analyses based on age (<60/≥60 years), sex (female/male), BMI (<25/25–30/≥30 kg/m^2^), hypertension (No/Yes), CHD (No/Yes), and CHF (No/Yes) were conducted to explore the correlation between METS-VF and DKD risk (Figure 3). The findings suggested a consistent relationship in the above subgroups, with no evidence of significant interactions (*p* for interaction > 0.05).

### 3.6. Mediating effect analysis

**Figure 4.**
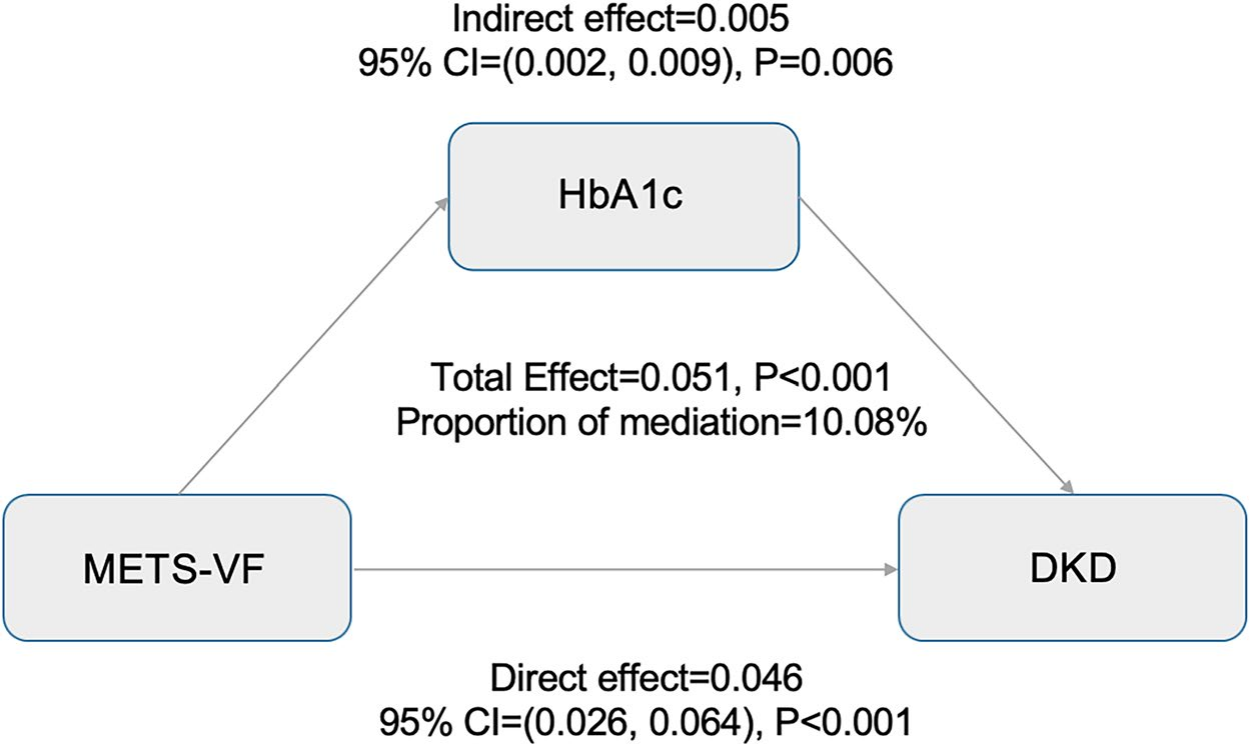
The results of mediating effect analysis.

Figure 4 presents the potential mediating effect of HbA1c on the relationship between METS-VF and DKD, with METS-VF defined as the independent variable, DKD as the dependent variable, and HbA1c functioning as the mediator. According to the Sobel test results, HbA1c served as a significant mediator in this relationship (*p* < 0.001). Notably, HbA1c contributed to 10.08% of the association between METS-VF and DKD (*p* < 0.001).

## 4. Discussion

To our knowledge, this is the pioneering study within the diabetic population investigating the link between METS-VF and the likelihood of DKD. The analysis demonstrates that METS-VF is an independent risk factor for DKD (*p* < 0.001). The METS-VF serves as a straightforward and potentially valuable epidemiological indicator for examining visceral fat’s contribution to DKD risk.

Recent investigations have reported associations between METS-VF and chronic kidney disease (CKD) risk in general populations [19–21]. However, our study provides distinct complementary insights by specifically targeting DKD in diabetic patients, who exhibit unique pathophysiological mechanisms compared to general CKD populations. We also identified a clinically actionable METS-VF threshold (7.42) for risk stratification and elucidated HbA1c as a significant mediator in the METS-VF-DKD pathway. These findings extend existing knowledge by offering diabetes-specific, mechanistically informed tools for precise DKD risk assessment in clinical practice. The challenge of the “obesity paradox” persists in epidemiological research, as the intricate nature of anthropometric data often hinders the precise identification of biologically driven disease risks [22–25]. Conventional obesity measures, such as BMI and WC, fail to distinguish body fat distribution effectively [23,26,27]. Visceral fat is a direct driver of metabolic disorders [28]. METS-VF offers a simple, noninvasive, and cost-effective alternative for assessing visceral fat and its related metabolic risks [29–30]. The METS-VF exceeds both traditional indices, such as BMI and WC, as well as composite indices like the visceral adiposity index (VAI) and weight-adjusted waist index (WWI), by also integrating insulin sensitivity and WHtR to better capture central obesity, while accounting for age and sex to provide a more individualized and accurate assessment [26,31]. According to our results of ROC and DCA, METS-VF also showed higher superiority than other traditional obesity indicators. On the other hand, METS-VF and DKD risk exhibit a J-shaped relationship: when METS-VF is low, the risk of DKD increases relatively slowly, reflecting the initial compensation of visceral fat [32–33]. Research has indicated that individuals with metabolically healthy obesity (MHO) exhibit no heightened susceptibility to cardiometabolic disorders, death, or CKD relative to those with normal weight [34]. An 8-year longitudinal Japanese investigation involving 3,136 subjects revealed that MHO participants showed no increased CKD incidence [35]. When contrasted with the metabolically healthy non-obese (MHNO) phenotype, the MHO cohort demonstrated an odds ratio of 0.83 for developing CKD [35]. Additionally, the observed increase in DKD risk at the lower end of the curve also reflects residual confounding by factors such as frailty or sarcopenia, conditions linked to both reduced adiposity and poor renal outcomes [36]. However, after the score surpasses the threshold, DKD risk rises sharply, which is closely associated with lipotoxicity, chronic inflammation, and insulin resistance caused by excessive visceral fat accumulation [32,37]. The threshold range of METS-VF may reflect the optimal metabolic balance of visceral fat. Clinically, METS-VF can be used as an indicator of metabolic health, and precise management to maintain it within an optimal range not only helps in controlling the risk of DKD. However, this threshold was statistically determined and lacks established clinical validation. Its applicability may be limited to our studied population. Therefore, caution is warranted in directly applying this finding in clinical practice, and future prospective studies in diverse cohorts are needed to confirm its value for DKD risk. We also observed that METS-VF correlates more strongly with reduced eGFR than with albuminuria. Notably, individuals with lower METS-VF may have lower muscle mass, which can result in an overestimation of eGFR when calculated using creatinine-based equations.

From biological mechanisms, visceral fat plays a significant role in the progression of DKD by contributing to insulin resistance, systemic inflammation, oxidative stress, and dysregulation of adipokines. It releases excessive free fatty acids that impair insulin signaling, causing chronic hyperglycemia, glomerular hyperfiltration, and renal damage [38–39]. Visceral fat acts as an endocrine organ secreting pro-inflammatory cytokines like TNF-α and IL-6, which exacerbate systemic inflammation, impair endothelial and podocyte function, and promote renal fibrosis [40–41]. Oxidative stress is heightened by the free fatty acids and cytokines, leading to mitochondrial overproduction of reactive oxygen species (ROS) that damage renal cells and disrupt nitric oxide (NO) balance, further driving glomerular scarring [42–43]. Furthermore, reduced adiponectin and elevated leptin and resistin in obesity disrupt glucose metabolism and activate profibrotic pathways (e.g., TGF-β and NF-κB), accelerating DKD progression [44].

METS-VF serves as a cost-effective tool to stratify DKD risk, particularly in settings without access to imaging. Its use of common clinical parameters facilitates rapid risk assessment and support early intervention. These findings have important implications for clinical practice, as the American Diabetes Association (ADA) and Kidney Disease: Improving Global Outcomes (KDIGO) guidelines emphasize the need for improved risk stratification tools in diabetic patients [45]. METS-VF demonstrated superior discriminative performance compared to traditional obesity measures and could be readily integrated into existing DKD risk assessment protocols. However, this study has certain limitations. Firstly, the cross-sectional design of this study cannot distinguish causal relationships between METS-VF and DKD. Secondly, while METS-VF offers a practical and noninvasive estimate of visceral fat, it lacks the accuracy provided by imaging tools such as CT or MRI. Validation of these findings through imaging-based studies is recommended. Thirdly, despite multivariate adjustments, the possibility of residual confounding remains. Important variables such as medication use (e.g., SGLT2 inhibitors, GLP-1 receptor agonists), diabetes duration, physical activity levels, and dietary habits were not included in our analysis. These unmeasured factors may influence both visceral fat and DKD risk, warranting careful consideration in future studies. Additionally, future research involving larger, multicenter, and longitudinal studies is necessary to establish causal relationships and evaluate the utility of METS-VF for predicting DKD progression over time.

## 5. Conclusion

Our study indicates that a higher METS-VF is linked to a greater risk of DKD. The METS-VF showed stronger discriminative power for DKD compared to other obesity indicators. However, further research is required to validate these findings.

## Data Availability

This study draws on data from the NHANES database, a publicly available resource that could be accessed at https://wwwn.cdc.gov/nchs/nhanes. Summary data and the corresponding R statistical code supporting these findings are available in the Figshare database at https://doi.org/10.6084/m9.figshare.29605889.
